# Fine Breakpoint Mapping by Genome Sequencing Reveals the First Large X Inversion Disrupting the *NHS* Gene in a Patient with Syndromic Cataracts

**DOI:** 10.3390/ijms222312713

**Published:** 2021-11-24

**Authors:** Alejandra Damián, Raluca Oancea Ionescu, Marta Rodríguez de Alba, Alejandra Tamayo, María José Trujillo-Tiebas, María Carmen Cotarelo-Pérez, Olga Pérez Rodríguez, Cristina Villaverde, Lorena de la Fuente, Raquel Romero, Gonzalo Núñez-Moreno, Pablo Mínguez, Carmen Ayuso, Marta Cortón

**Affiliations:** 1 Department of Genetics & Genomics, Instituto de Investigación Sanitaria-Fundación Jiménez Díaz University Hospital, Universidad Autónoma de Madrid (IIS-FJD, UAM), 28040 Madrid, Spain; alejandra.damian@quironsalud.es (A.D.); mrodrigueza@fjd.es (M.R.d.A.); alejandra.tamayo@quironsalud.es (A.T.); mjtrujillo@fjd.es (M.J.T.-T); cvillaverde@fjd.es (C.V.); lorena.fuente@quironsalud.es (L.d.l.F.); raquel.romerof@quironsalud.es (R.R.); gonzalo.nunezm@quironsalud.es (G.N.-M); pablo.minguez@quironsalud.es (P.M.); cayuso@fjd.es (C.A.); 2 Centre for Biomedical Network Research on Rare Diseases (CIBERER), 28290 Madrid, Spain; 3 Department of Medical Genetics, University Hospital Clínico San Carlos, 28040 Madrid, Spain; raluca.oancea@salud.madrid.org (R.O.I.); mariacarmen.cotarelo@salud.madrid.org (M.C.C.P.); 4 Department of Pediatrics, University Hospital Clínico San Carlos, 28040 Madrid, Spain; oprodriguez@salud.madrid.org; 5 Bioinformatics Unit, Instituto de Investigación Sanitaria-Fundación Jiménez Díaz University Hospital, Universidad Autónoma de Madrid (IIS-FJD, UAM), 28040, Madrid, Spain

**Keywords:** congenital cataract, pericentric X inversion, *NHS*, Nance-Horan syndrome, structural variation (SV), whole-genome sequencing (WGS), chromosomal rearrangement, topologically associated domains (TADs), position effects

## Abstract

Inversions are structural variants that are generally balanced. However, they could lead to gene disruptions or have positional effects leading to diseases. Mutations in the *NHS* gene cause Nance-Horan syndrome, an X-linked disorder characterised by congenital cataracts and dental anomalies. Here, we aimed to characterise a balanced pericentric inversion X(p22q27), maternally inherited, in a child with syndromic bilateral cataracts by breakpoint mapping using whole-genome sequencing (WGS). 30× Illumina paired-end WGS was performed in the proband, and breakpoints were confirmed by Sanger sequencing. EdU assays and FISH analysis were used to assess skewed X-inactivation patterns. RNA expression of involved genes in the breakpoint boundaries was evaluated by droplet-digital PCR. We defined the breakpoint position of the inversion at Xp22.13, with a 15 bp deletion, disrupting the unusually large intron 1 of the canonical *NHS* isoform, and also perturbing topologically-associated domains (TADs). Moreover, a microhomology region of 5 bp was found on both sides. RNA analysis confirmed null and reduced *NHS* expression in the proband and his unaffected mother, respectively. In conclusion, we report the first chromosomal inversion disrupting *NHS*, fine-mapped by WGS. Our data expand the clinical spectrum and the pathogenic mechanisms underlying the *NHS* defects.

## 1. Introduction

Chromosomal structural variations (SVs) are a predominant cause of rare diseases [1]. SVs can be defined as genetic changes in the chromosome structure or DNA dosage, typically involving DNA segments that are larger than 1 kb [1,2,3]. Unbalanced rearrangements such as deletions or duplications are usually referred to as copy number variations (CNVs) that imply quantity changes in the genomic dosage [2,3]. Balanced rearrangements such as copy-number neutral insertions, inversions, or reciprocal translocations do not lead to any apparent loss or gain of genetic material [2,3]. An inversion arises when there are two breaks within the same chromosome, and the central DNA segment between both breaks rejoins in a reverse direction. As a result, the orientation of the genomic fragment is changed [4,5]. Inversions emerge from different processes of DNA recombination, reparation, or replication. These mechanisms often involve loops and crossovers between highly identical inverted genomic repeats at both breakpoint’s sides [4,6,7,8,9,10]. Pericentric inversions, whereby the inverted region within both chromosome arms includes the centromere, are the most common cytogenetically visible rearrangements in humans [11,12]. Frequent pericentric inversions reported in chromosomes 1, 2, 3, 5, 9, 10, and 16 involve only heterochromatic DNA [13]. In particular, a pericentric inversion of chromosome 9 is found with a frequency of 1–2% in the general human population [14]. In general, inversions have been traditionally considered neutral and usually appear to have no apparent phenotypic consequences apart from fertility effects due to an increased risk for miscarriages [4,14].

Excluding these common cytogenetically characterised polymorphisms, current knowledge about the possible clinical consequences of other rare chromosomal inversions has been largely overlooked. However, growing evidence has revealed an underestimated role of inversions in disease development by directly affecting gene expression in different ways [2,3,15]. Inversion breakpoints can lead to gene disruptions on their linear structure or alter the 3D structure of the implicated regions through positional effects [3,15]. Chromatin folding is organised in the nucleus with a 3D genome structure into topologically associating domains (TADs) [16,17]. Therefore, inversions disrupting TADs might separate genes from their regulatory elements or provide new regulatory sequences in a different regulatory domain, leading to gene deregulations with phenotypic consequences [2,3,15,17].

Accurate breakpoint determination is essential to unveil the pathogenic mechanisms underlying balanced inversions in patients with rare developmental diseases [15,18,19]. Unfortunately, fine-mapping of SVs at single-nucleotide resolution is challenging in routine clinical procedures and, accordingly, is rarely performed. Generally, their detection relies on standard cytogenetics techniques that have several drawbacks. Karyotyping is limited to the detection of rearrangements involving more than 5–10 Mb. Fluorescence in situ hybridisation (FISH) could reach higher resolutions of up to 100 kb, but is limited to the targeted probes used [20]. Alternative molecular approaches based on array comparative genome hybridisation (array-CGH) efficiently detect subchromosomal CNVs of around ~20–200 kb, but balanced SVs remain undetectable [20,21]. The increasing use of exome sequencing to identify causal variants has not improved the characterisation of these balanced SVs due to its inability to detect breakpoints, usually located in non-coding regions. Therefore, the actual contribution of inversions and other balanced SVs as disease-causing variants is currently underestimated [4].

Recent breakthroughs in genomic sequencing raised new approaches to characterise the exact breakpoint positions for SVs at a reasonable cost. Indeed, different studies on large cohorts of patients carrying balanced SVs showed the advantage of conducting short-read whole-genome sequencing (WGS) to detect efficiently chromosomal rearrangements [4,15,22].

This work aimed to pinpoint a balanced pericentric inversion in chromosome X found in a boy suffering from a syndromic form of congenital cataracts. After his cytogenetic detection, this maternally inherited inversion was initially considered non-pathogenic and non-causal, and other genetic causes were further discarded. Using WGS, we established that this X-inversion transcriptionally disrupts the gene responsible for the Nance-Horan syndrome (NHS), a rare X-linked syndromic disorder mainly characterised by a variable expressivity of congenital cataracts, dental anomalies, and other features [23,24,25]. Then, short-read WGS, gene expression, and X-inactivation analysis were applied to decipher the contribution of this X inversion in the phenotypic expression of cataracts and other clinical features in this family.

## 2. Results

### 2.1. Clinical Features

**Figure 1:**
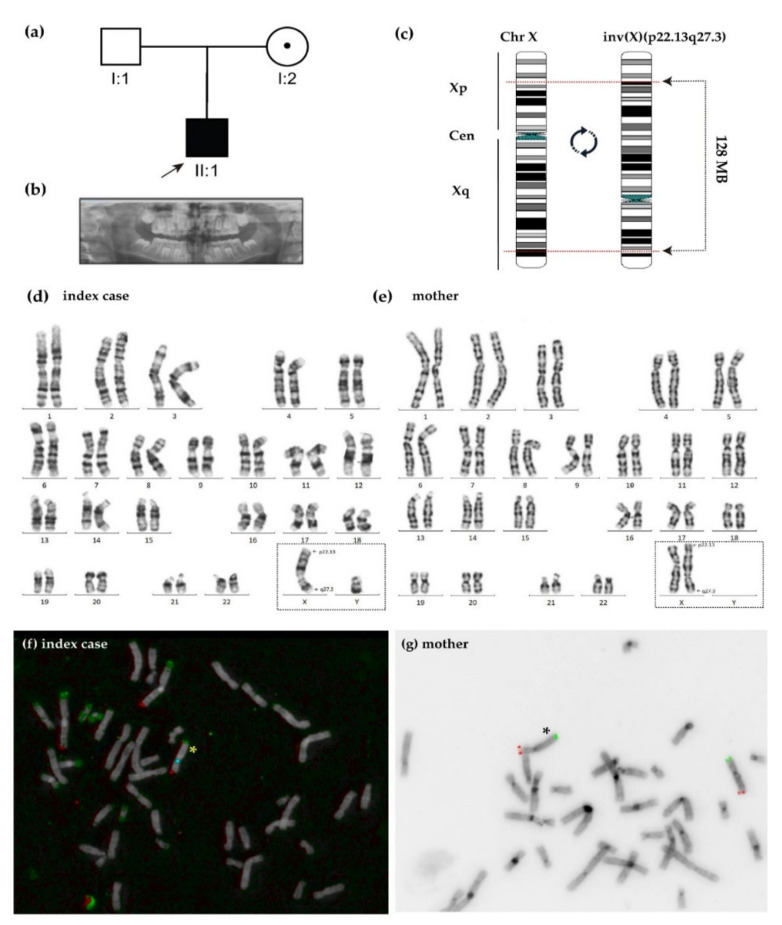
(**a**) Familiar pedigree of the index case with syndromic bilateral cataracts including dental anomalies and supernumerary teeth in orthopantomography (**b**). Black boxes denote affected male boy, dotted circle, carrier healthy mother, clear boxes, unaffected father. (**c**–**g**) Karyotyping and FISH analysis revealed a large and balanced pericentric inversion X(p22q27) in the index case (**d**,**f**) and his mother (**e**,**g**). Xp/Yp, Xq/Yq, and CEP X probe (locus DxZ1) are shown in green, red, and blue, respectively. Yellow or black asterisks show the X-inverted chromosome.

The index case (II:1, Figure 1a) is a 10-year-old male who presented bilateral congenital cataracts at birth. He was the first child of a healthy non-consanguineous couple of Colombian ancestry with no familial history of congenital cataracts or other genetic disorders. His mother (I:2) was asymptomatic by the time her son was diagnosed with congenital cataracts. In addition, the boy also presented dysmorphic features, developmental motor delay, Chiari malformation, hypercalcemia, ventricular septal defect, and seizures at the first year of age. Later on, the boy started to have dental pains at the age of eight. After an orthopantomography (Figure 1b), supernumerary teeth and other dental anomalies were observed in the index case. A summary of the clinical features of the patient is shown in Table A1.

### 2.2. Genetic Analysis

Karyotyping in the index case identified a balanced pericentric inversion on the X chromosome, 46 Y, inv(X)(p22q27), maternally inherited (Figure 1c–e). This finding was confirmed by fluorescence in situ hybridisation (FISH) (Figure 1f–g). No gain or loss of genetic material was detected by using a high-resolution 180 K array CGH; thus, it was defined as a copy-number neutral inversion.

Subsequent analysis of single-nucleotide variants (SNVs) and CNVs was performed by clinical exome sequencing of a 243-gene virtual panel associated with ocular developmental diseases (ODDs), which includes 63 genes related to pediatric cataracts. No pathogenic variants were found that could explain the patient’s phenotype.

**Figure 2:**
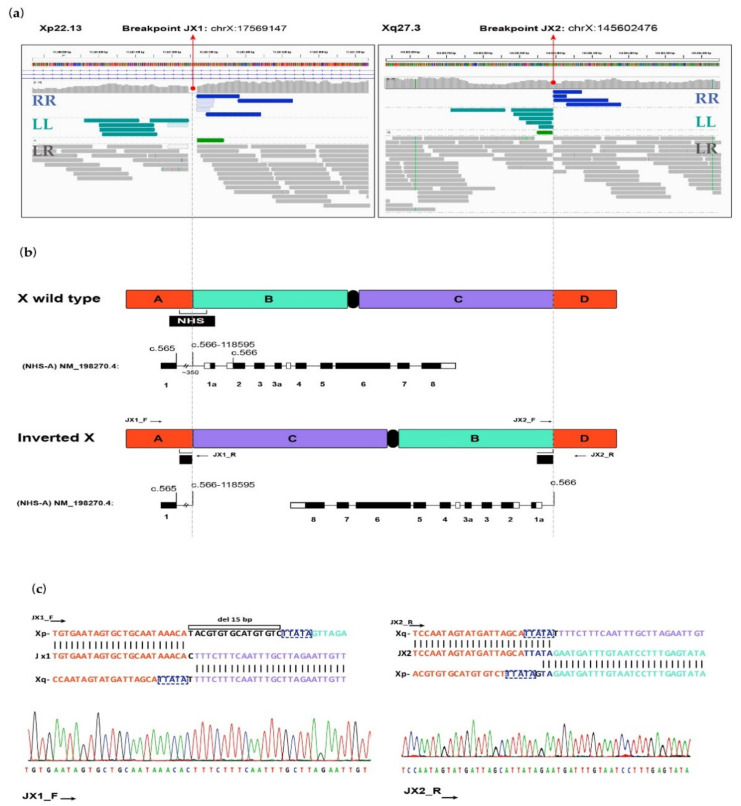
Fine mapping of the balanced pericentric large inversion Inv (X)(p22.13q27.3) by whole genome sequencing. (**a**) Integrative Genomics Viewer (IGV) screenshot of the genomic regions containing the proximal JX1 breakpoint at Xp22.13, disrupting the intron 1 of *NHS*, and the distal JX2 breakpoint at an intergenic region of Xq27.3 of the 128-Mb inversion identified in the index case. Gene, read coverage, and alignment tracks are shown from top to bottom. Reads are grouped and coloured by pair orientation. Normal left-right (LR) paired reads are coloured in grey. Discordant reads with unexpected pair orientation indicating a structural variant are coloured in green representing read pairs with left-left (LL) pair orientation, and in blue, read pairs with right-right (RR) pair orientation. A 15-bp deletion was identified in the proximal breakpoint. (**b**) Schematic representation of the rearrangement on the X-chromosome, fine mapping of breakpoints and disruption of the canonical NHS-A isoform by the inversion in JX1. The colour of the chromosome blocks indicates the inverted sequence on the Xp arm (green), and the inverted sequence on the Xq arm (purple). The vertical lines highlight the JX1 and JX2 breakpoint junctions. (**c**) Fine mapping of breakpoint junctions by Sanger sequencing. The background colours correspond to the scheme shown in (**b**), deleted 15-bp sequence in JX1 is coloured in black, and the 5-bp microhomology sequence in JX1 and JX2 is in dark blue.

The index case was subjected to a paired-end WGS analysis with an average coverage of 30X. As reflected in Figure 2a, a 128 Mb pericentric inversion on Xp22.13q27.3 was correctly called that changes the chromosomal position of the *NHS locus*, involved in an X-linked rare syndromic form of congenital cataracts, the Nance-Horan syndrome (MIM #302350).

The precise position of the breakpoints at the nucleotide level resolution was also fine-mapped (Figure 2b–c). The first breakpoint at Xp22.13, and hereafter referred to as JX1, was located at chrX:g.17569147, disrupting the *NHS* gene in the middle of the intron 1 of the main transcriptional isoform *NHS*-A (NM_198270.4) (Figure 2b). In addition to the inversion, a small deletion of 15 nucleotides at chrX:g.17569124-17569141 (hg38) was found in the JX1 junction. The second breakpoint, at Xq27.3, hereafter referred to as JX2, was located at chrX:g.145602476. This breakpoint position does not disrupt any genes, and it is within an intergenic region. Furthermore, Sanger sequencing of the PCR-amplified specific fragment junctions confirmed both breakpoints involving JX1 and JX2 and the 15-bp deletion (Figure 2c). Analysis of the nucleotide sequence of both junctions revealed a common region of microhomology of 5 bp TTATA. The index case and her mother were shown to be hemizygous and heterozygous carriers, respectively, for this rearranged SV.

### 2.3. Correlation Phenotype—Genotype

Classical Nance-Horan syndrome is an X-linked disorder, mainly characterised by the association of congenital bilateral cataracts, dental anomalies, and dysmorphic features, as summarised in Table A1. This syndrome shows high penetrance but variable expressivity among males and females carrying *NHS* mutations. In the family under study, a complete presentation of the classical Nance-Horan syndrome is found only in the index case, who shows additional minor signs previously reported in this syndrome. Our patient has a slight global developmental delay (45% visual disability and 10% cognitive and motor impairment). The developmental delay has been reported only in one-third of the male patients suffering from Nance-Horan syndrome. Congenital cardiac malformations are rarely presented in some NHS patients. Our patient also has an unusual presentation of infantile seizures at the first year of age.

**Figure A1:**
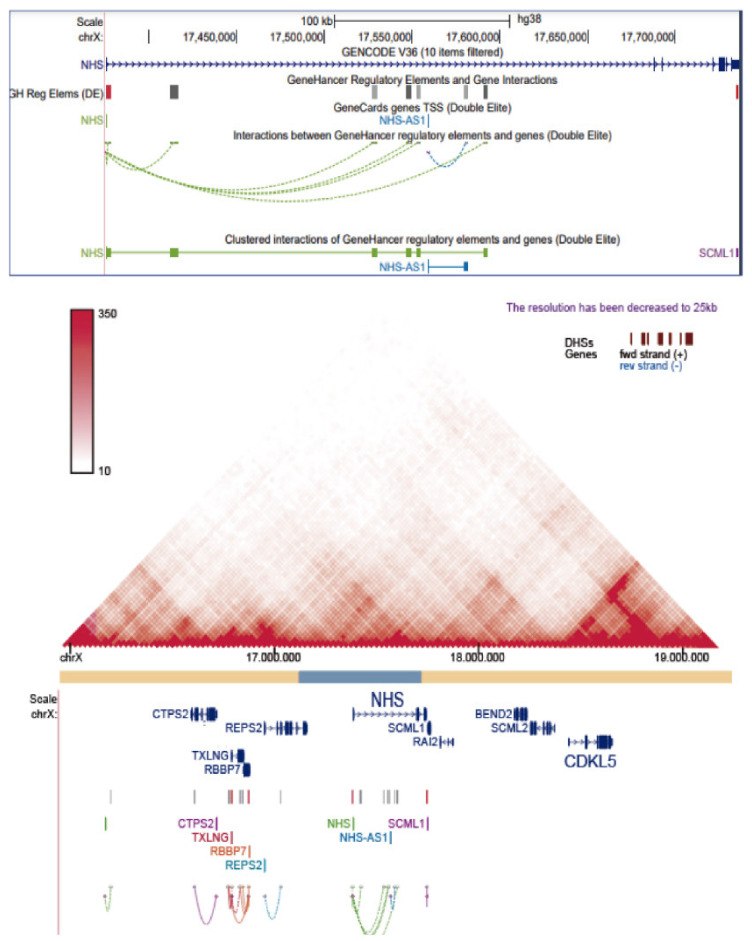
Three-dimensional (3D)-genome map at the Xp22.13. Public Hi-C data from a B-Lymphocyte cell line (GM12878) was used. Breakpoint disrupts TADs boundaries, and gene interactions between regulatory elements on the *NHS* gene (in blue box).

We hypothesised if the inversion would influence the phenotype with a possible transcriptional dysregulation of the *NHS* gene or other nearby genes. First, using publicly available Hi-C data from the lymphocyte cell line GM12878, the analysis of the three-dimensional (3D)-genome map at both breakpoint junctions, showed the disruption of TADs in the Xp22.13 *locus*. In this position, the interactions between regulatory elements located on the side of the proximal JX1 breakpoint junction and nearest regions are disrupted (Figure A1). Here, the *NHS* gene, which spans 360.5 kb on chromosome Xp22.13, is alternatively spliced in at least described five isoforms (*NHS*-1A, A–D). Of them, the *NHS*-A transcript that contains an alternative exon 1 and an unusual large intron 1 of around 30 kb, is disrupted by the distal breakpoint of the X inversion in our patient (Figure 2b and Figure A1). However, the transcriptional regulation of the other *NHS* isoforms that are not intersected by the breakpoint, may also be potentially affected due to disruption of TADs. Additionally, *CDKL5*, a gene associated with developmental and epileptic encephalopathy (MIM #300672), is located ~0.7 Mb apart from the JX1 breakpoint, thus seems a good candidate to contribute to the phenotype of the patient. In the JX2 junction on the locus Xq27.3, the breakpoint has no consequence on regulatory elements (data not shown).

### 2.4. Transcriptional Analysis

**Figure 3:**
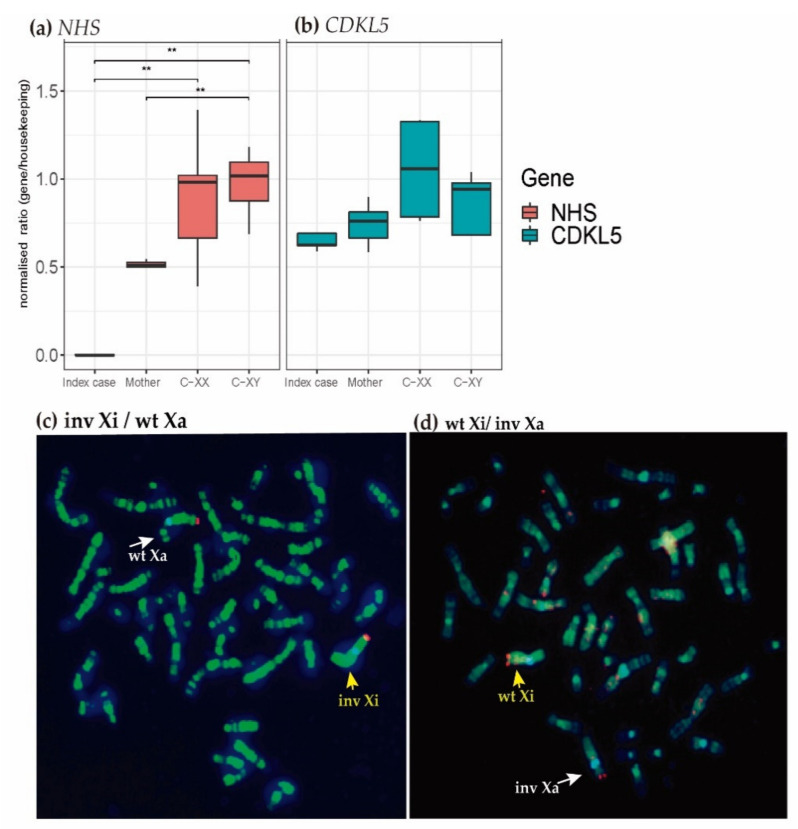
Functional characterisation of the inversion. (**a**,**b**) Boxplots represent the relative mRNA expression of *NHS* (**a**) and *CDKL5* (**b**) in blood for the index case and his mother compared to 3 males and 4 females healthy controls. Expression of the housekeeping *UB2DE2* gene was used for setting the relative expression. The experiment was performed in triplicates. Boxplot indicates median values and whiskers range of variation. ** = *p* < 0.01 (Wilcoxon test). C-XX: Female controls; C-XY: male controls. (**c**–**d**) X-chromosome inactivation pattern by EdU incorporation assay in lymphocyte cultures from the mother. X-chromosomes marked in total full green fluorescence reflect late replication associated with X-chromosome inactivation (yellow arrows). The X chromosome weakly banding labelled in green indicates active X-chromosomes (white arrows). Metaphases from the mother show: (**c**) inactive inverted X-chromosome (Inv-Xi) and active wild type X-chromosome (wt-Xa) and (**d**) inactive wild type X-chromosome (wt-Xi) and active inverted X-chromosome (Inv-Xa). Note: Edu incorporation was labelled with Alexa Fluor-488 and counterstained with DAPI. The X-centromeres were marked in blue in Cyan blue (CEPX probe) and the Xq telomeres in red (Telvysion Xq/Yq probe).

To delve into the possibility of transcriptional deregulations caused by the inversion at the JX1 breakpoint, we have assessed the relative mRNA expression in blood of the isoforms *NHS*-A, *NHS*-1A, *NHS*-B, *NHS*-C, and *NHS*-D, as well as the nearby *CDKL5* gene for the index patient and his mother and compared them with sex-matched healthy controls. Droplet digital PCR (ddPCR)-based gene expression analysis showed null expression normalised ratios of *NHS* (mean fold-change (FC) = 0.003, *p*-value < 0.005) in the index case and a half downregulation in his mother (FC = 0.5, *p*-value < 0.005) compared with healthy female and male controls, respectively (Figure 3a). By contrast, no evidence of differential expression was observed for *CDKL5* (Figure 3b).

### 2.5. X-Chromosome Inactivation (XCI) Analysis

To further understand the differential expression of the X-inversion, the possible existence of non-random patterns of X-chromosome inactivation (XCI) in the mother was investigated. A HUMARA assay, based on analysis of methylated sequences of the Androgen Receptor gene at Xq11.2, was uninformative due to homozygosity for the short tandem repeats (STRs) analysed. We then used an alternative method to detect late replication of inactive X-chromosomes based on the fluorescent EdU (5-ethynyl-2′-deoxyuridine) incorporation on blood cell cultures. After counting a total of 279 metaphases, we observed a similar XCI pattern for the inverted and the wild-type X-chromosomes: an XCI ratio of 0.58 (162/279) for inactive inverted *versus* active wildtype X-chromosomes and an XCI ratio of 0.42 (117/279) for inactive wild-type *versus* active inverted X-chromosomes (Figure 3c). The confidence interval was between 0.53–0.63, below the upper limit of 0.70, indicating random inactivation and no skewed pattern. Thus, this assay indicated that only 58% of the mother’s blood cells had inactivation of the inverted X-chromosome (Figure 3c–d), so it was considered non-skewed. In brief, both X chromosomes are randomly inactivated, and no skewing is found in the mother.

## 3. Discussion

We present here the first large novel X pericentric inversion associated with the disruption of the *NHS* gene that is causing the Nance-Horan syndrome. Balanced SVs, in which no apparent gain or loss of chromatin is involved, are undetected by the most molecular approaches that are now widely implemented in routine genetic diagnosis. Exome sequencing only targets exons; thus, 99% of genomic regions remain unscreened. Array-CGH only detects CNVs using targeted approaches; thus, balanced SVs cannot be resolved [15,20]. In addition, when identified through classical G-banded chromosome analysis, balanced inversions are traditionally considered likely benign, therefore dismissed as disease causes [15].

With the advent of WGS approaches, there is increasing evidence that some SVs can cause disease phenotypes due to gene disruption, cryptic copy-number imbalance, or gene dysregulation by positional effects [3,16,26]. Paired-end WGS mapping of SVs using the algorithm of read-pair orientation has been efficiently used in detecting breakpoints in highly repetitive regions such as centromeric regions, segmental duplications, and short arms [15,18,19,27,28,29,30,31]. Using this strategy, we successfully identified breakpoints at a base-pair resolution of a large balanced inversion, first characterised as inv(X)(p22q27) in a male patient suffering from a syndromic form of congenital cataracts; thus, providing a more precise clinical interpretation for this SVs.

Breakpoint positions were efficiently detected, corresponding to a simple inversion rearrangement with two chromosome arms breaks. Several mechanisms could be involved in creating chromosomal inversions as non-allelic homologous recombination (NAHR). However, they can also be originated by non-homologous end joining (NHEJ) or replication-based mechanisms mediated by microhomology, such as fork stalling and template switching (FoSTeS), and microhomology-mediated break-induced replication (MMBIR) [4,7,8,9,10,23]. At the junctions of the two genomic segments that contributed to the X inversion, a sequence of microhomology of five identical nucleotides, TTATA, and a small deletion of 15 nucleotides in the proximal breakpoint was found. This pattern of microhomology with deletions of >10 nucleotides is highly indicative of the implication of MMBIR as the mechanism responsible for the formation of this inversion [8].

Only the proximal breakpoint disrupting the *NHS* gene seemed to contribute to the patient’s phenotype. This junction lies on the *NHS*-A RNA isoform, where exon 1 is taken apart for the rest of the 3′ *NHS*-associated exons. *NHS-A* is considered the main responsible of the pathogenesis [32]. Also, this particularly large alternative intron 1 of 30 kb seemed to be part of the regulatory machinery of *NHS* [23,25,33]. Inversions and other SVs can also disrupt TADs on the 3D chromatin, which are more prone to interact with each other [1,16,26,34,35,36]. The disruption of TAD boundary integrity can contribute to the transcriptional dysregulation of nearby genes [37].

Our analysis also revealed that the inversion could disrupt the integrity of TADs and regulatory elements located on the proximal JX1 junction, potentially affecting the gene regulation of *NHS* and other genes. The *NHS* gene, highly conserved across vertebrate species, regulates actin remodeling and cell morphology during development in the midbrain, retina, lens and tooth [24,37]. Previous studies have shown that this ubiquitously expressed gene is completely absent in the blood samples of Nance-Horan patients [33,38]. Gene expression assays confirmed the total lack of expression for the major isoform *NHS*-A in the boy, supporting a loss of *NHS* function caused by the X-inversion here described. Besides, alternative *NHS* B–D transcripts, harbouring an alternative exon/intron1, were silenced in the hemizygous patient, although these isoforms remain intact in the inverted segment. Thus, not-yet-defined regulatory transcriptional elements located in the large intron 1 of the *NHS* gene seem necessary for expressing the three alternative isoforms *NHS* B–D. Therefore, our findings support the hypothesis that positional effects are seemingly involved in the pathogenesis of this inversion through the physical separation of regulatory elements of the *NHS* gene.

Nance-Horan syndrome is a rare X-linked disorder whose prevalence remains unknown, as no more than 50 families have been reported to date. The mutational database HGMD (last accession on 31 July 2021) has described 73 *NHS* mutations, mainly loss-of-function variants and CNVs: 20 nonsense, 8 missense, four splicing mutations, one regulatory variant, and 38 small and gross indels. Nevertheless, few complex rearrangements involving the *NHS locus* have also been described, including intragenic chromosomal rearrangements [23] and a balanced translocation X;1 that disrupts *NHS* [38].

Clinical findings described in our patient were bilateral cataracts, dental anomalies, dysmorphic features, and developmental delay, typically present in the classical Nance Horan phenotype. However, some grades of variable expressivity have been reported for other uncommon symptoms only reported in a few patients (Table A1). Significant variability in terms of intellectual disability has been described, which suggests a possible modulation effect of other genetic factors [39]. Our patienthas a developmental delay and other very infrequent features for this syndrome, such as cardiac anomalies [23] and an unusual presentation of seizures. Other patients with contiguous gene deletions affecting the *NHS* gene also present atypical Nance-Horan syndrome [38,39,40]. Moreover, the implication of a possible role of flanking genes on NHS expressivity in patients with CNVs has been also speculated [40]. For instance, a hemizygous *de novo* 2.8-Mb microdeletion Xp22.2–Xp22.13 was described, including the *CDKL5* and *NHS* genes, both contributing to the associated phenotypic presentation of severe encephalopathy, congenital cataracts, and tetralogy of Fallot in a 10-month-old boy [41].

To delve deeper into the potential role of the flanking genes of the X-inversion breakpoints that could be transcriptionally modulated by positional effects, gene expression analysis was carried out. This analysis was critical to clarify the potential contribution of the nearby gene *CDKL5*, which is the main responsible for atypical Rett syndrome with early-onset seizures. A positional effect also potentially leading to *CDKL5* deregulation, was thought to explain the atypical neurological features observed in our patient. However, the *CDKL5* expression in blood was similar in both carriers of this X inversion compared with a sex-matched control group, which seems to discard its implication in the phenotypic presentation of our patient. We assessed other potential contributors to the patient phenotype on both proximal and distal breakpoints. However, the potential effect on the phenotype of other nearby genes to the breakpoints, is even less evident. None of them has been associated with a human phenotype or OMIM disease. On Xp22.13, *SCLM1* is likely involved in spermatogenesis, with a potential role in azoospermia [40], *SCLM2* seem to participate in embryonic development and *RAI2* is a retinoic acid-induced gene that may play a role in cardiogenesis, for which a potential implication on cardiac anomalies in Nance-Horan syndrome has been previously speculated [23,33,39,40,41]. On Xq27.3, the breakpoint is situated in an intergenic region between *SPANXN1* and *SLITRK2*, around 350 kb and 220 kb away from the junction, respectively. *SPANXN1* play a role in spermiogenesis [42] and *SLITRK2* may modulate neurite activity [43]. In addition, the gene expression of all these potentially affected genes could not be assessed in our patient because preliminary analysis showed very low or no levels in blood. Therefore, the contribution of these genes to our patient’s phenotype remains unclear.

Variable disease expressivity in female carriers has been reported in X-linked disorders such as the Nance-Horan syndrome [23,25,38,44]. Usually, this is caused by skewed inactivation of an X chromosome carrying SVs or other genetic defects [37]. X chromosome inactivation usually occurs randomly in each female cell to turn off one of the two copies of the X chromosome [38,44]. Preferential inactivation of the aberrant X chromosome generally impairs the phenotypic expression. This syndrome is fully expressed in males, although heterozygous carrier females may develop certain features, usually milder cataracts in their 40s [23,38,44,45]. In the family here presented, the mother did neither present congenital cataracts nor additional symptoms. However, after the diagnosis of Nance-Horan syndrome in her son, a recent ophthalmic revision in the 3rd decade of life showed subclinical light lens opacities. To investigate if the phenotypic variability in this family could be related to any X chromosome’s skewed inactivation, the X inactivation patterns were measured in the carrier mother. The traditional HUMARA method for XCI analysis [46] was not informative due to the homozygosity of the studied STRs in this *locus*. Thus, an alternative strategy for XCI was performed based on the EdU incorporation into proliferating cells [47]. Since EdU is a labelled nucleoside analogue of thymidine, its incorporation during DNA synthesis can reveal replication-associated active and inactive regions on the X chromosomes. This assay showed random XCI in the carrier mother in blood with no differences of inactivation in the inverted and the wild-type X chromosome. Considering all the data, the observed random inactivation of one X chromosome in the carrier mother could explain the half downregulation of the transcriptional *NHS* levels.

To conclude, in this work, we describe the first large balanced pericentric inversion inv(X)(p22.13q27.3) as the cause of syndromic cataracts in a boy with an overlapping presentation of classical Nance-Horan syndrome. First, short paired-end WGS allowed us to efficiently characterise the breakpoint junctions at a nucleotide level, revealing the disruption of the linear structure of the main *NHS* isoform in an alternative intron 1. Moreover, the genomic 3D structure of the *NHS* gene is also altered, implicating TADs and potential regulatory elements. Further expression analysis confirmed the complete lack of *NHS* expression. Therefore, a positional effect seems also to be involved in the pathogenesis of the Nance-Horan syndrome through silencing other *NHS* transcripts whose coding regions are not affected by the inverted sequences. Thus, our findings expand the mutational mechanisms and phenotypic spectrum underlying this syndrome. Finally, this work points out the importance of breakpoint fine-mapping of copy-number neutral rearrangements to improve diagnostic yield in undiagnosed Mendelian disorders.

## 4. Materials and Methods

### 4.1. Cytogenetic Analysis

Karyotyping and FISH analysis were performed on the boy and his mother using commercial FISH probes (CEPX & Telvysion Xp/Yp, Xq/Yq, Abbott Molecular, Des Plaines, IL, USA) following standard procedures.

A 5-ethynyl-2′-deoxyuridine (Edu) incorporation assay was designed to assess the X-inactivation pattern (Click-iT™ EdU Cell Proliferation Kit for Imaging, Alexa Fluor™ 488 dye, Invitrogen, Waltham, MA, USA). Lymphocytes from heparinised peripheral blood were first treated with 10 μM EdU for a total of 4 h at 37 °C, and then, incubated with 0.1ml of ColcemidTM (Gibco, Waltham, MA, USA) for 90 min, at 37 °C, followed by 0.56% KCl treatment for 8 min, at 37 °C, and finally treated with Carnoy fixative (3:1 methanol: acetic acid). EdU-treated chromosomes were dropped onto clean glass slides. According to the manufacturer’s protocol, the FISH assay was performed using CEPX & Telvysion Xq/Yq probes (Abbott Molecular). The slides were denatured at 73 °C for 5 min and incubated at 37 °C for 24 h. EdU incorporation was detected by a copper-catalysed reaction between the alkyne group of EdU and the azide group of Alexa Fluor^®^ 488 dye. Finally, the chromosomes were counterstained with DAPI (Abbott Molecular). The metaphases were analysed under a fluorescence microscope, and images were captured using the ISIS program (MetaSystems Hard & Software, Altlussheim, Germany). A total of 200 metaphases were analysed. Statistical analysis was performed to assess the ratio of X inactivation.

### 4.2. Molecular Analysis

Genomic DNA was isolated from whole peripheral blood using an automated DNA extractor. Array-CGH was performed using a previously custom design to target 150 ODD-related genes (SurePrint G3 4 × 180 k CGH array, Agilent Technologies, Santa Clara, CA, USA), including 19,165 probes for the X chromosome [48]. Array-CGH was analysed using the Agilent CytoGenomics software v.2.7 with default analysis methods.

For clinical exome sequencing, coding exons of 4813 known genes associated with inherited diseases were captured using the commercial TruSightOne Sequencing Panel kit (Illumina, San Diego, CA, USA). Paired-end (2 × 150) libraries were sequenced in an Illumina NextSeq500 platform. Bioinformatic analyses were performed using the Illumina BaseSpace and Variant Studio software, respectively. Variants were prioritised by a subpanel of 143 ODDs genes, including 63 cataract-associated genes.

For WGS, the library was prepared from 1 µg of genomic DNA using the NEB Next^®^ Ultra™ DNA Library Prep Kit (New England Biolab, Ipswich, MA, USA) and sequenced by paired-end (2 × 150 bp) in an Illumina Novaseq6000 platform at 30× coverage. Raw sequencing reads were aligned to the GRCh38/hg38 assembly using the BWAv0.7.15 with default parameters. Split reads at the potential proximal Xp22 and distal Xq27 breakpoints junctions of the inversion were visualised on the genome reference using the Integrative Genomics Viewer (IGV) to identify discordant paired-reads with unexpected pair orientation.

The sequences flanking the exact breakpoints were compared for similarities to each other and to repeat elements using UCSC BLAT and RepeatMasker, respectively. TADs that could be potentially disrupted on the inversion boundaries were searched on previous Hi-C data (GM12878 cells Rao_2014_raw [49]), available on 3D Genome Browser (http://3dgenome.fsm.northwestern.edu/view.php; last accession on 25 May 2021) using GeneHancer Regulatory Elements [50] and ENCODE Gene Interactions and Integrated Regulation tracks.

Breakpoint junctions were validated by PCR and subsequently by Sanger sequencing using specific flanking primers for the proximal JX1 junction on Xp22.13 and the distal JX2 on Xq27.3 (Table A2).

### 4.3. RNA Expression Analysis

Total RNA from whole peripheral blood of the index patient, his mother, and seven healthy controls (four females and three males) was isolated with the NucleoSpin RNA Blood kit (Macherey-Nagel, Allentown, PA, USA). RT-PCR was conducted using random primers with the SuperScript™ IV Reverse Transcriptase (Invitrogen) to synthesise full-length cDNA. cDNA was fluorometrically quantified in a Qubit™ Flex Fluorometer with the dsDNA HS Assay Kit (Thermo Fisher Scientific, Waltham, MA, USA). Relative expression analysis was performed in triplicate by ddPCR using the QX200 ddPCR EvaGreen^®^ Supermix (Bio-Rad, Hercules, CA, USA) according to manufacturer’s protocol, and specific primers (Table A2) for *NHS*, *CDKL5* and four endogenous gene housekeeping controls (*GAPDH*, *RPS18*, *UB2DE2,* and *ACTB).* Following droplet generation using Oil for EvaGreen^®^ into the QX200 Droplet Generator, emulsion PCR was amplified on a C1000 Touch Thermal Cycler (Bio-Rad) under the following conditions: 95 °C for 10 min, 40 cycles of PCR at 94 °C for 30 s, and 57 °C for 1 min, and a final step at 98 °C for 10 min. ddPCR data were analysed with the Quantasoft v1.7.4 software (Bio-Rad) using absolute quantification settings. Ratios of the absolute quantity of cDNA per sample (expressed as copies/μL) were normalised using the two more homogeneous housekeeping genes, *RPS18* and *UB2DE2*, between all samples. The non-parametric Wilcoxon signed-rank test was used to assess the statistical significance between different ratios of the groups.

## Data Availability

All data relevant to the study are included in the article.

## Appendix A

**Table A1:** Clinical features of the index case correlated to mayor and minor signs of the Nance-Horan syndrome.

Clinical Symptoms	Index Case
**Mayor signs in 80–99% of NHS patients**	
Cataract	Yes
Long face	No
Mandibular prognathia	Yes
Microcornea	Yes
Nystagmus	Yes
Prominent nasal bridge	Yes
Prominent nose	Yes
Visual loss	Yes
**Signs in 30–79% of NHS patients**	
Increased number of teeth	Yes
Intellectual disability	Yes
Protruding ear	Yes
Short metacarpal	No
Strabismus	Yes
Behavioral abnormality	No
Glaucoma	No
Microphthalmia	No
Retinal detachment	No
**Minor signs in < 4% of NHS patients**	
Intellectual disability, moderate	Yes
Mulberry molar	Unknown
Screwdriver-shaped incisors	No
**Additional minor symptoms**	
Autism	No
Broad finger	No
Developmental cataract	Yes
Diastema	No
Macrotia	Yes
Narrow face	No
Posterior Y-sutural cataract	Unknown
Short phalanx of finger	No
Supernumerary maxillary incisor	Yes
X-linked dominant inheritance	Yes
Abnormal heart morphology	Yes
Abnormality of the urogenital system	Yes
Seizures	Yes

**Table A2:** PCR and RT-PCR primers.

Primers	Gene	Exons	Size (BP)	Sequence (5′–3′)
JX1_DNA_F	JX1 fragment		454	GAGAAAATGCGGTGTTTGGT
JX1_DNA_R				GGGAGATTTCAACACCCCACT
JX2_DNA_F	JX2 fragment		542	AGGTGCTGGAGAGGATGTGG
JX2_DNA_R				TTGAGGTTGCTTTGCTCTTG
NHS_RNA_F	*NHS*	E6-E7	99	GAGACCCAAGGAAATGTGGA
NHS_RNA_R				TGCTTCAGCTTGGGAGTCTT
CDKL5_RNA_F	*CDKL5*	E4-E5	101	ACATGAAATTGTGGCGATCA
CDKL5_RNA_R				GCTTGAGAGTCCGAAGCATT
GAPDH_RNA_F	*GADPH*	E8-E9	203	CGACCACTTTGTCAAGCTCA
GAPDH_RNA_R				AGGGGAGATTCAGTGTGGTG
RPS18_RNA_F	*RPS18*	E2–E3	130	GTTCCAGCATATTTTGCGAGT
RPS18_RNA_R				GTCAATGTCTGCTTTCCTCAAC
ACTB_RNA_F	*ACTB*	E1–E2	110	ACAGAGCCTCGCCTTTG
ACTB_RNA_R				CCTTGCACATGCCGGAG
UBE2D2_RNA_F	*UBE2D2*	E7–E8	120	GTACTCTTGTCCATCTGTTCTCTG
UBE2D2_RNA_R				CCATTCCCGAGCTATTCTGTT

## References

[1] Sudmant P.H., Rausch T., Gardner E.J., Handsaker R.E., Abyzov A., Huddleston J., Zhang Y., Ye K., Jun G., The 1000 Genomes Project Consortium (2015). An integrated map of structural variation in 2,504 human genomes. Nature.

[2] Feuk L., Carson A.R., Scherer S.W. (2006). Structural variation in the human genome. Nat. Rev. Genet..

[3] Spielmann M., Lupiáñez D.G., Mundlos S. (2018). Structural variation in the 3D genome. Nat. Rev. Genet..

[4] Pettersson M., Grochowski C.M., Wincent J., Eisfeldt J., Breman A.M., Cheung S.W., Krepischi A.C.V., Rosenberg C., Lupski J.R., Ottosson J. (2020). Cytogenetically visible inversions are formed by multiple molecular mechanisms. Hum. Mutat..

[5] Giner-Delgado C., Villatoro S., Lerga-Jaso J., Gayà-Vidal M., Oliva M., Castellano D., Pantano L., Bitarello B.D., Izquierdo D., Noguera I. (2019). Evolutionary and functional impact of common polymorphic inversions in the human genome. Nat. Commun..

[6] Puig M., Casillas S., Villatoro S., Cáceres M. (2015). Human inversions and their functional consequences. Brief. Funct. Genom..

[7] Koumbaris G., Hatzisevastou-Loukidou H., Alexandrou A., Ioannides M., Christodoulou C., Fitzgerald T., Rajan D., Clayton S., Kitsiou-Tzeli S., Vermeesch J.R. (2011). FoSTeS, MMBIR and NAHR at the human proximal Xp region and the mechanisms of human Xq isochromosome formation. Hum. Mol. Genet..

[8] Ottaviani D., LeCain M., Sheer D. (2014). The role of microhomology in genomic structural variation. Trends Genet..

[9] Lee J.A., Carvalho C.M.B., Lupski J.R. (2007). A DNA replication mechanism for generating nonrecurrent rearrangements associated with genomic disorders. Cell.

[10] Weckselblatt B., Rudd M.K. (2015). Human structural variation: Mechanisms of chromosome rearrangements. Trends Genet..

[11] Feuk L. (2010). Inversion variants in the human genome: Role in disease and genome architecture. Genome. Med..

[12] Schmidt S., Claussen U., Liehr T., Weise A. (2005). Evolution versus constitution: Differences in chromosomal inversion. Hum. Genet..

[13] Liehr T., Weise A., Mrasek K., Ziegler M., Padutsch N., Wilhelm K., Al-Rikabi A. (2019). Recombinant chromosomes resulting from parental pericentric inversions—Two new cases and a review of the literature. Front. Genet..

[14] Sismani C., Rapti S.-M., Iliopoulou P., Spring A., Neroutsou R., Lagou M., Robola M., Tsitsopoulos E., Kousoulidou L., Alexandrou A. (2020). Novel pericentric inversion Inv(9)(P23q22.3) in unrelated individuals with fertility problems in the Southeast European population. J. Hum. Genet..

[15] Schluth-Bolard C., Diguet F., Chatron N., Rollat-Farnier P.-A., Bardel C., Afenjar A., Amblard F., Amiel J., Blesson S., Callier P. (2019). Whole genome paired-end sequencing elucidates functional and phenotypic consequences of balanced chromosomal rearrangement in patients with developmental disorders. J. Med. Genet..

[16] McArthur E., Capra J.A. (2021). Topologically associating domain boundaries that are stable across diverse cell types are evolutionarily constrained and enriched for heritability. Am. J. Hum. Genet..

[17] Shanta O., Noor A., Sebat J., Human genome structural variation consortium (HGSVC) (2020). The effects of common structural variants on 3D chromatin structure. BMC Genom..

[18] Suzuki T., Tsurusaki Y., Nakashima M., Miyake N., Saitsu H., Takeda S., Matsumoto N. (2014). Precise detection of chromosomal translocation or inversion breakpoints by whole-genome sequencing. J. Hum. Genet..

[19] Sanchis-Juan A., Stephens J., French C.E., Gleadall N., Mégy K., Penkett C., Shamardina O., Stirrups K., Delon I., Dewhurst E. (2018). Complex structural variants in mendelian disorders: Identification and breakpoint resolution using short- and long-read genome sequencing. Genome Med..

[20] Silva M., de Leeuw N., Mann K., Schuring-Blom H., Morgan S., Giardino D., Rack K., Hastings R. (2019). European Guidelines for Constitutional Cytogenomic Analysis. Eur. J. Hum. Genet..

[21] Pös O., Radvanszky J., Buglyó G., Pös Z., Rusnakova D., Nagy B., Szemes T. (2021). Copy number variation: Characteristics, evolutionary and pathological aspects. Biomed. J..

[22] Lindstrand A., Eisfeldt J., Pettersson M., Carvalho C.M.B., Kvarnung M., Grigelioniene G., Anderlid B.-M., Bjerin O., Gustavsson P., Hammarsjö A. (2019). From cytogenetics to cytogenomics: Whole-genome sequencing as a first-line test comprehensively captures the diverse spectrum of disease-causing genetic variation underlying intellectual disability. Genome Med..

[23] Coccia M., Brooks S.P., Webb T.R., Christodoulou K., Wozniak I.O., Murday V., Balicki M., Yee H.A., Wangensteen T., Riise R. (2009). X-linked cataract and Nance-Horan syndrome are allelic disorders. Hum. Mol. Genet..

[24] Brooks S.P. (2004). Identification of the gene for nance-horan syndrome (NHS). J. Med. Genet..

[25] Burdon K.P., McKay J.D., Sale M.M., Russell-Eggitt I.M., Mackey D.A., Wirth M.G., Elder J.E., Nicoll A., Clarke M.P., FitzGerald L.M. (2003). Mutations in a novel gene, NHS, cause the pleiotropic effects of Nance-Horan syndrome, including severe congenital cataract, dental anomalies, and mental retardation. Am. J. Hum. Genet..

[26] Norton H.K., Phillips-Cremins J.E. (2017). Crossed wires: 3D genome misfolding in human disease. J. Cell Biol..

[27] Plesser Duvdevani M., Pettersson M., Eisfeldt J., Avraham O., Dagan J., Frumkin A., Lupski J.R., Lindstrand A., Harel T. (2020). Whole-genome sequencing reveals complex chromosome rearrangement disrupting *NIPBL* in infant with Cornelia de Lange syndrome. Am. J. Med. Genet..

[28] Wu Z., Wu Y., Gao J. (2020). InvBFM: Finding genomic inversions from high-throughput sequence data based on feature mining. BMC Genom..

[29] Vuillaume M.-L., Cogné B., Jeanne M., Boland A., Ung D.-C., Quinquis D., Besnard T., Deleuze J.-F., Redon R., Bézieau S. (2018). Whole genome sequencing identifies a de NOVO 2.1 Mb balanced paracentric inversion disrupting FOXP1 and leading to severe intellectual disability. Clinica Chimica Acta.

[30] Pons L., Bouvagnet P., Bakloul M., Di Filippo S., Buisson A., Chatron N., Labalme A., Metton O., Mitchell J., Diguet F. (2019). Supravalvular aortic stenosis caused by a familial chromosome 7 inversion disrupting the ***ELN*** gene uncovered by whole-genome sequencing. Mol. Syndromol..

[31] Spiegler S., Rath M., Hoffjan S., Dammann P., Sure U., Pagenstecher A., Strom T., Felbor U. (2018). First large genomic inversion in familial cerebral cavernous malformation identified by whole genome sequencing. Neurogenetics.

[32] Shoshany N., Avni I., Morad Y., Weiner C., Einan-Lifshitz A., Pras E. (2017). NHS gene mutations in Ashkenazi Jewish families with Nance–Horan syndrome. null.

[33] Liao H.-M., Niu D.-M., Chen Y.-J., Fang J.-S., Chen S.-J., Chen C.-H. (2011). Identification of a microdeletion at Xp22.13 in a Taiwanese family presenting with Nance-Horan syndrome. J. Hum. Genet..

[34] Melo U.S., Schöpflin R., Acuna-Hidalgo R., Mensah M.A., Fischer-Zirnsak B., Holtgrewe M., Klever M.-K., Türkmen S., Heinrich V., Pluym I.D. (2020). Hi-C identifies complex genomic rearrangements and TAD-shuffling in developmental diseases. Am. J. Hum. Genet..

[35] Van Bemmel J.G., Galupa R., Gard C., Servant N., Picard C., Davies J., Szempruch A.J., Zhan Y., Żylicz J.J., Nora E.P. (2019). The bipartite TAD organization of the X-inactivation center ensures opposing developmental regulation of Tsix and Xist. Nat. Genet..

[36] Tokoro M., Tamura S., Suzuki N., Kakihara M., Hattori Y., Odaira K., Suzuki S., Takagi A., Katsumi A., Hayakawa F. (2020). Aberrant X chromosomal rearrangement through multi-step template switching during sister chromatid formation in a patient with severe hemophilia A. Mol. Genet. Genomic. Med..

[37] Brooks S.P., Coccia M., Tang H.R., Kanuga N., Machesky L.M., Bailly M., Cheetham M.E., Hardcastle A.J. (2010). The nance–horan syndrome protein encodes a functional WAVE homology domain (WHD) and is important for Co-ordinating actin remodelling and maintaining cell morphology. Hum. Mol. Genet..

[38] Gómez-Laguna L., Martínez-Herrera A., Reyes-de la Rosa A.D.P., García-Delgado C., Nieto-Martínez K., Fernández-Ramírez F., Valderrama-Atayupanqui T.Y., Morales-Jiménez A.B., Villa-Morales J., Kofman S. (2018). Nance–Horan syndrome in females due to a balanced X;1 translocation that disrupts the *NHS* gene: Familial case report and review of the literature. Ophthalmic Genet..

[39] Accogli A., Traverso M., Madia F., Bellini T., Vari M.S., Pinto F., Capra V. (2017). A novel Xp22.13 microdeletion in Nance-Horan syndrome: Xp22.13 microdeletion in NHS. Birth Defects Res..

[40] Milunsky A., Milunsky J.M., Dong W., Hovhannisyan H., Oates R.D. (2020). A contiguous microdeletion syndrome at Xp23.13 with non-obstructive azoospermia and congenital cataracts. J. Assist. Reprod. Genet..

[41] Van Esch H., Jansen A., Bauters M., Froyen G., Fryns J.-P. (2007). Encephalopathy and bilateral cataract in a boy with an interstitial deletion of Xp22 comprising the CDKL5 and NHS genes. Am. J. Med. Genet..

[42] Westbrook V.A., Schoppee P.D., Vanage G.R., Klotz K.L., Diekman A.B., Flickinger C.J., Coppola M.A., Herr J.C. (2006). hominoid-specific SPANXA/D genes demonstrate differential expression in individuals and protein localization to a distinct nuclear envelope domain during spermatid morphogenesis. Mol. Hum. Reprod..

[43] Aruga J., Mikoshiba K. (2003). Identification and characterization of Slitrk, a novel neuronal transmembrane protein family controlling neurite outgrowth. Mol. Cell. Neurosci..

[44] Khan A.O., Aldahmesh M.A., Mohamed J.Y., Alkuraya F.S. (2012). Phenotype-genotype correlation in potential female carriers of x-linked developmental cataract (Nance-Horan syndrome). Ophthalmic Genet..

[45] Migeon B.R. (2020). X-linked diseases: Susceptible females. Genet. Med..

[46] Allen R.C., Zoghbi H.Y., Moseley I.A.B., Rosenblatt H.M., Belmont J.W. (1992). Methylation of Hpall and Hhal sites near the polymorphic CAG repeat in the human androgen-receptor gene correlates with X chromosome inactivation. Am. J. Hum. Genet..

[47] Sisdelli L., Vidi A.C., Moysés-Oliveira M., Di Battista A., Bortolai A., Moretti-Ferreira D., Dias da Silva M.R., Melaragno M.I., Carvalheira G. (2016). Incorporation of 5-ethynyl-2′-deoxyuridine (EdU) as a novel strategy for identification of the skewed X inactivation pattern in balanced and unbalanced X-rearrangements. Hum. Genet..

[48] Ceroni F., Aguilera-Garcia D., Chassaing N., Bax D.A., Blanco-Kelly F., Ramos P., Tarilonte M., Villaverde C., da Silva L.R.J., Ballesta-Martínez M.J. (2019). New GJA8 variants and phenotypes highlight its critical role in a broad spectrum of eye anomalies. Hum. Genet..

[49] Rao S.S.P., Huntley M.H., Durand N.C., Stamenova E.K., Bochkov I.D., Robinson J.T., Sanborn A.L., Machol I., Omer A.D., Lander E.S. (2014). A 3D map of the human genome at kilobase resolution reveals principles of chromatin looping. Cell.

[50] Fishilevich S., Nudel R., Rappaport N., Hadar R., Plaschkes I., Iny Stein T., Rosen N., Kohn A., Twik M., Safran M. (2017). GeneHancer: Genome-wide integration of enhancers and target genes in GeneCards. Database.

